# Characterization of Molecular Interactions in the Bondline of Composites from Plasma-Treated Aluminum and Wood

**DOI:** 10.3390/molecules28227574

**Published:** 2023-11-14

**Authors:** Sascha Jan Zimmermann, Philipp Moritz, Oliver Höfft, Lienhard Wegewitz, Wolfgang Maus-Friedrichs, Sebastian Dahle

**Affiliations:** 1 Clausthal Center for Materials Technology, Clausthal University of Technology, Agricolastrasse 2, 38678 Clausthal-Zellerfeld, Germany; 2 Institute for Electrochemistry, Clausthal University of Technology, Arnold-Sommerfeld-Strasse 6, 38678 Clausthal-Zellerfeld, Germany; 3 Department of Wood Science and Technology, Biotechnical Faculty, University of Ljubljana, Jamnikarjeva Ulica 101, 1000 Ljubljana, Slovenia; sebastian.dahle@bf.uni-lj.si

**Keywords:** spin coating, thin films, DBD plasma, PVAc, hydrogen bonding, wood, interface

## Abstract

Wood and aluminum composites are becoming increasingly attractive due to their ability to combine the advantages of both materials: the lightweight nature of wood and the strength of aluminum. However, using conventional wood adhesives like polyvinyl acetate (PVAc) to bond these dissimilar materials is challenging and requires special surface treatments. Prior studies have demonstrated that applying a dielectric barrier discharge plasma treatment significantly enhances shear and bending strengths in beech wood/aluminum bonds. This study focuses on the molecular interactions between PVAc and aluminum or beech wood influenced by plasma surface modification. Surface-sensitive methods, including X-ray photoelectron spectroscopy, infrared reflection adsorption spectroscopy and atomic force microscopy, were employed to characterize the PVAc films on the corresponding surfaces and to identify possible interactions. The ultrathin PVAc films required for this purpose were deposited by spin coating on untreated and plasma-treated aluminum. The aluminum surface was cleaned and oxidized by plasma. Additionally, hydroxyl species could be detected on the surface. This can lead to the formation of hydrogen bonds between the aluminum and the carbonyl oxygen of PVAc after plasma treatment, presumably resulting in increased bond strength. Furthermore, the beech wood surface is activated with polar oxygen species.

## 1. Introduction

**Figure 1:**
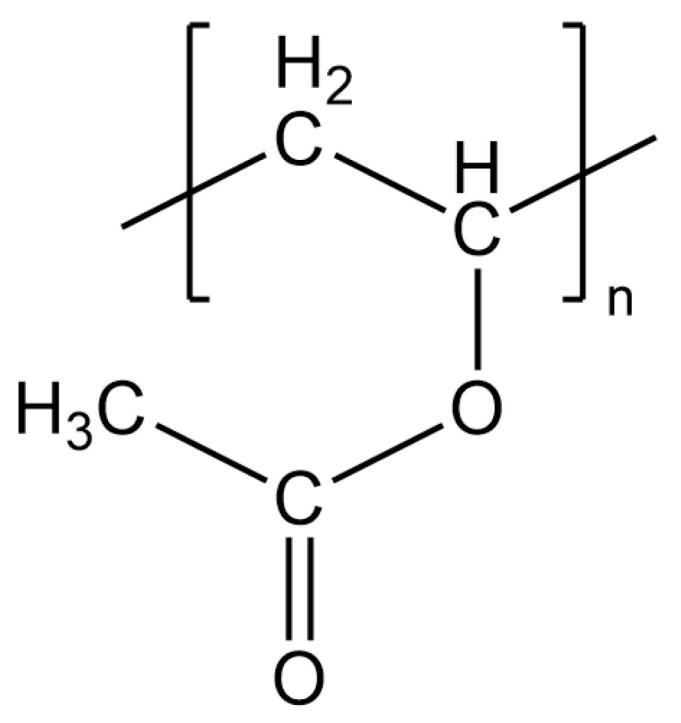
Structural formula of the polyvinyl acetate monomer unit.

The composite of wood and aluminum combines the properties of wood, such as its low density, thermal insulation, durability and renewability [1,2,3], with the properties of aluminum, such as mechanical stability and corrosion resistance [4]. A particular challenge is the environmentally friendly joining of these two materials [5,6]. Primarily, adhesives are used to join such dissimilar materials. Despite their enormous importance, especially in the 21st century, the molecular interactions of adhesives with the surfaces are not yet fully understood [7,8]. Adhesives are characterized primarily by their functional groups, which can interact with the surface and with other adhesive molecules. Polyvinyl acetate (PVAc) is one of these adhesives, and its monomer unit is shown in Figure 1. This polymer has a high adhesion strength to wood, so it is mainly used as a wood adhesive [9,10] but leads to lower strengths with other materials such as aluminum [11].

To improve the performance of such bonded materials, there are many studies that focus on the chemical modification of the adhesive [12,13]. On the other hand, the metal surface can also be modified to improve the adhesive properties. Therefore, Žigon et al. [14] determined the strengths of metal–wood bonds for different adhesives. Furthermore, the materials were treated with plasma before bonding. This results in an increase in shear strength, especially beech wood and aluminum bonded with PVAc increased by 69.5% in shear force due to the prior plasma treatment. Further studies showed an even larger increase in shear strength and bending strength, as well as an impact on the modulus of elasticity of the compound. Notably, the PVAc bonds of plasma-treated substrates outperformed state-of-the-art adhesives for such composites as epoxy and polyurethane [15]. However, the question remains open: what modifications occur on the surfaces due to the plasma treatment, i.e., what interactions at the molecular level lead to this significant increase in shear strength?

In this study, we used a dielectric barrier discharge (DBD) plasma for the surface modification of beech wood and aluminum. To achieve comparability, the same materials were used as in the previously mentioned studies by Žigon et al. The plasma is ignited between two electrodes, with the aluminum substrate serving as one separated by a dielectric barrier. During the treatment with plasma, ions, electrons, radicals and UV radiation are formed, which interact with the surface and potentially clean it from impurities or activate it with functional groups [16]. DBD plasma offers advantages over chemical processes, such as control, stability, reproducibility and the elimination of vacuum technology [17,18,19]. X-ray photoelectron spectroscopy (XPS) and infrared reflection adsorption spectroscopy (IRRAS) are used to track the changes at the molecular level due to plasma treatment. XPS uses X-rays to obtain information about the chemical composition and binding states of the sample surface [20]. IRRAS uses polarized IR light to obtain information about molecular changes due to adsorption of thin organic films on a reflective metal surface [21]. For both methods, the presence of ultrathin PVAc films of a few nanometers is essential. For polymers such as polymethyl methacrylate (PMMA) or polyethyl cyanoacrylate (PECA), spin coating has already been tested by the authors in earlier works as a suitable method for producing such thin films [22,23]. Furthermore, atomic force microscopy (AFM) is used to investigate the topography to determine whether it is a closed and homogeneous adhesive film. The aim of this work is to explain the increase in shear strength between aluminum and beech wood by plasma treatment on a molecular level by detecting the existing intermolecular and interatomic interactions.

## 2. Results

### 2.1. Comparison of Untreated with Plasma-Treated Beech Wood

**Figure 2:**
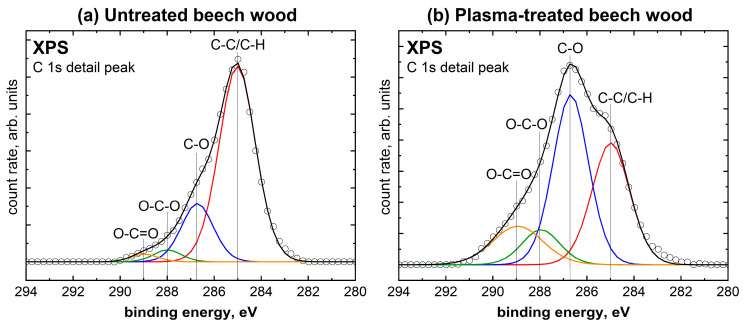
XPS C 1s detail spectra of (**a**) untreated beech wood and (**b**) plasma-treated beech wood. For the graph shown, the enveloping black line represents the sum of the peak fit while the circles reflect the measured values.

In order to detect changes on the surface of beech wood due to plasma treatment, XPS spectra were recorded for untreated and plasma-treated wood. The corresponding survey spectra are included in the Supporting Information. The C 1s detail spectra are shown in Figure 2. The ratio of oxygen to carbon was determined from the XPS spectra to be 0.32 for untreated beech wood. A 10 s plasma treatment of the wood sample more than doubled the oxygen/carbon ratio to 0.74. A similar oxidizing effect can be observed in other studies on plasma treatment of wood [24,25]. Figure 2 shows a typical C 1s detail spectrum for beech wood. It contains the species aliphatic C-C/C-H at 285.0 eV, C-O at 286.7 eV, O-C-O at 288 eV, and O-C=O at 288.97 eV [26]. As can be seen in Table 1, the species C-C/C-H decreases by more than half because of the plasma treatment. In contrast, all proportions of the oxygen-containing species are increasing. Accordingly, the C 1s detail spectrum shows significantly more oxygen bound to carbon and, thus, explains the change in the oxygen/carbon ratio. The O-C=O species show the largest plasma-induced increase. Nevertheless, the proportions of all three oxygen-containing species belonging to the polar functional groups increased significantly. These polar functional groups on the surface can lead to much better wetting [27], especially with polar liquids, and should lead to better surface adhesion properties [28]. Accordingly, it is assumed that plasma treatment of the wood leads to potentially better adhesion between PVAc and beech wood.

### 2.2. Comparison of Untreated with Plasma-Treated Aluminum Samples

**Figure 3:**
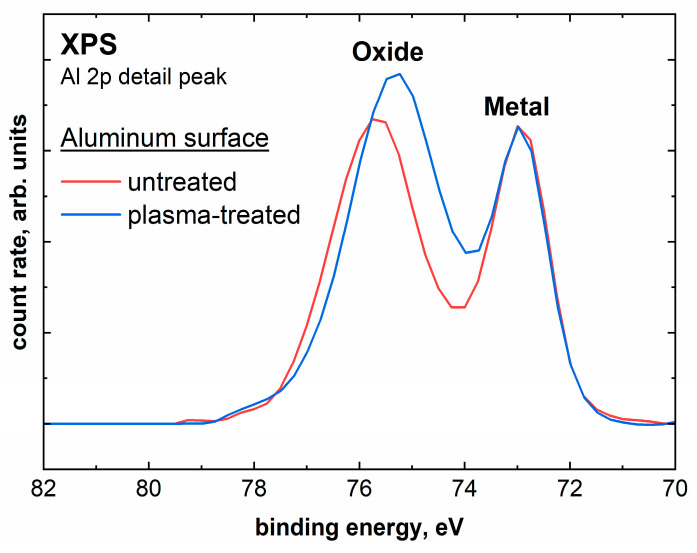
XPS Al 2p detail spectra of untreated and plasma-treated polished aluminum. The intensity is normalized to the metallic peak at 73 eV.

In order to detect changes on the polished aluminum surface, XPS spectra were recorded for both untreated and plasma-treated samples. Figure 3 shows the Al 2p detail spectra for the untreated and plasma-treated aluminum samples. The Al 2p peak is particularly suitable for evaluation, as a distinction can be made between metallic aluminum and other components such as oxides [29]. The metallic aluminum can be assigned to the peak at 73 eV, to which the intensity was normalized in order to compare the measurements. The second peak at higher binding energies between 75 eV and 76 eV can be assigned to various oxygen species, such as aluminum oxides or hydroxides [29]. The separation and differentiation of the individual oxide peaks are hindered by the use of non-monochromatic radiation [29]. The metal/oxide intensity ratio of the untreated sample is 0.62. After a 10 s plasma treatment under atmospheric air, this ratio changes to 0.46. Accordingly, there is significantly more oxygen detectable in the surface region of the aluminum sample. Furthermore, a peak shift in the oxide peak towards the lower binding energy due to the plasma treatment can be seen in Figure 3. This effect can be explained by the presence of additional hydroxyl species on the surface of the sample [30]. The stoichiometry also changes significantly due to the plasma treatment. The aluminum concentration decreases from 38.1 at.% to 34.5 at.% after the plasma treatment, while the oxygen concentration increases from 50.7 at.% to 57.6 at.%. Furthermore, 2.3 at.% nitrogen could be determined after the plasma treatment. The increase in oxide and hydroxyl components on the aluminum surface (see Figure S3) increases the wettability of the aluminum [31]. Moreover, the hydroxyl groups are beneficial for possible interactions with the adhesive.

**Figure 4:**
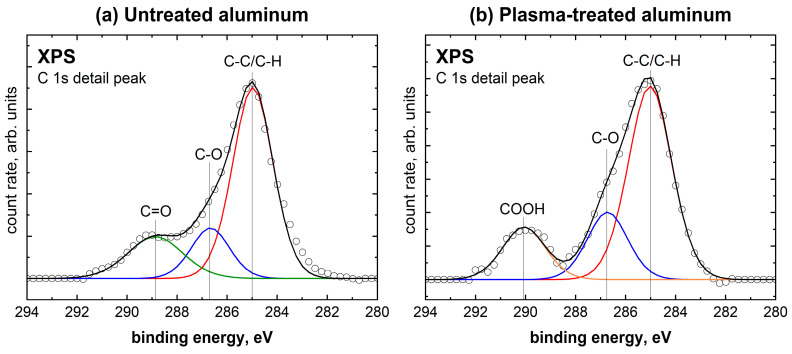
XPS C 1s detail spectra of (**a**) untreated aluminum and (**b**) plasma-treated aluminum.

Furthermore, the effect of the plasma treatment on the carbonaceous impurities on the aluminum surface was also investigated using XPS. A change in stoichiometry was observed, with the carbon content dropping from 8.7 at.% to 5.4 at.% as a result of the plasma treatment. This indicates a surface-cleaning effect through plasma, which has also been observed in other studies on aluminum. [32,33]. The species present in the C 1s detail peak, which can be seen in Figure 4, are C-C/C-H at 285.0 eV, C-O at 286.7 eV, C=O at 288.9 eV, and COOH at 290 eV. As seen in Table 2, the untreated aluminum sample shows aliphatic carbon, with further proportions of C-O and C=O groups. After the plasma treatment, the C=O species could no longer be detected, but a new species of COOH was formed at 290 eV. Accordingly, the C=O will be oxidized to COOH through plasma treatment [34].

**Figure 5:**
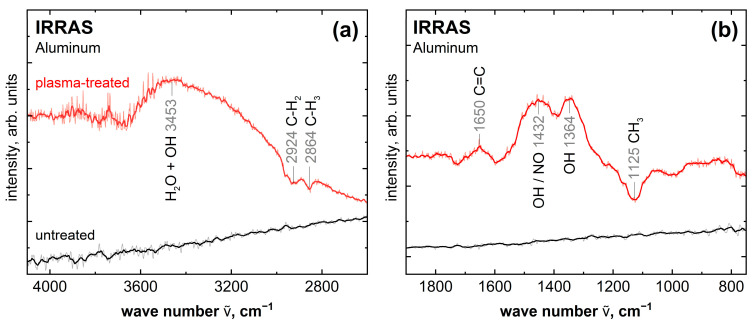
IRRAS spectra of plasma-treated (red) and untreated (black) aluminum. (**a**) 4100 cm^−1^ to 2500 cm^−1^ and (**b**) 1900 cm^−1^ to 700 cm^−1^. Shown are the measured data (light color) and the moving average of 12 measured points (dark color).

The spectra of the IRRAS measurements, which can be seen in Figure 5, show the changes on the surface of the plasma-treated aluminum sample compared to the untreated reference. Here, the same sample was first measured as a background spectrum and then measured again. In between is the plasma treatment in one case. The subtraction of the first from the second measurement results in the spectra shown. Peaks in the negative direction indicate a decrease and positive peaks an increase in the corresponding species. The untreated surface shows a featureless spectrum, indicating that no new functional groups are created here between the measurements. The plasma-treated aluminum surface, on the other hand, shows clear changes. The negative peaks at 2924 cm^−1^, 2864 cm^−1^ and 1125 cm^−1^ are assigned to the species C-H_2_ and C-H_3_ [35]. Accordingly, IRRAS also shows the already observed cleaning effect of the aluminum sample through plasma treatment. Furthermore, a peak could be detected at 1650 cm^−1^, which can be assigned to the species C=C [35]. This indicates a transformation of the carbon impurities on the aluminum sample through plasma. The peaks at 3453 cm^−1^ and 1364 cm^−1^ are associated with hydroxyl and water species, respectively [35,36]. The hydroxyl bands can explain the observed peak shift in the Al 2p measurement, seen in Figure 3, of the plasma-treated aluminum sample. The peak at 1432 cm^−1^ can be assigned to hydroxyl or NO species [36]. The species NO, which is probably direct-bonded to the aluminum, is supported by the XPS measurements since 2.3 at.% nitrogen was measured in the survey and detail spectrum after plasma treatment in atmospheric air (see Figures S2, S3 and S4 in Supporting Information). These OH and NO species are formed on the surface by the reaction of the aluminum surface with radical or reactive species in the plasma. The hydroxyl species are formed, for example, by the dissociation of absorbed water and by reactions with atomic oxygen, OH and H_2_O in the plasma [37]. The hydroxyl species increase the wettability by increasing the polar component of the surface energy [38].

### 2.3. Measurements of the PVAc Reference

**Figure 6:**
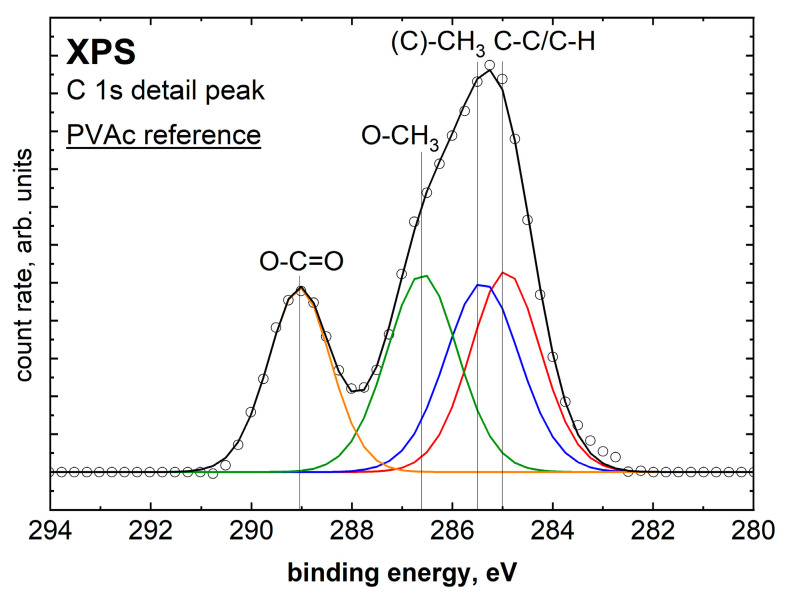
XPS C 1s detail peak of a 20 nm thick PVAc film on aluminum.

To identify any bonding mechanisms of thin films, a 20 nm thick PVAc film was analyzed as a reference. Figure 6 shows the XPS C 1s detail spectrum of a thick PVAc film on aluminum. The 20 nm film thickness was chosen as this is above the information depth of XPS and ensures that the aluminum sample and the interface between the sample and film do not affect the measurements. The C 1s detail spectrum shows a total of four species. The C 1s detail spectrum can be approximated with a combination of four binding types [39], as seen in Table 3.

The C 1s detail spectrum shows an approximately equal distribution of the individual species, which is consistent with the structural formula in Figure 1. 

**Figure 7:**
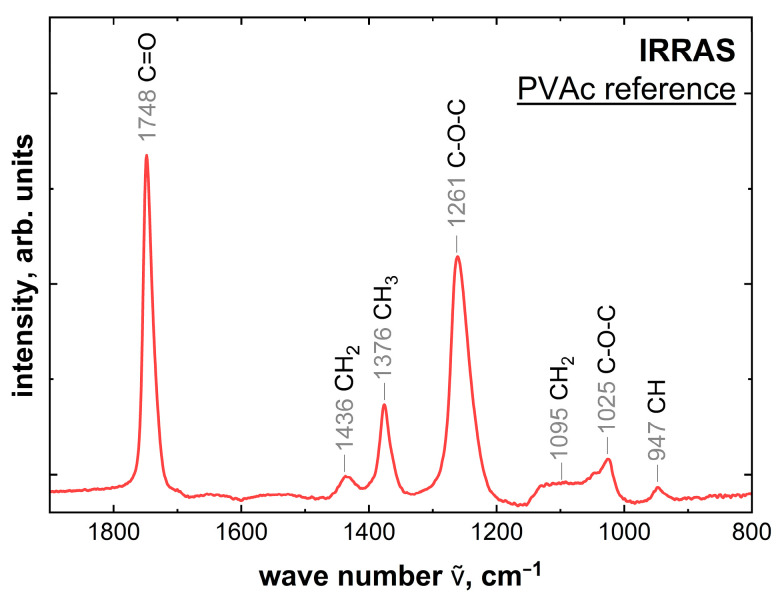
IRRAS spectrum of a 20 nm thick PVAc film on aluminum.

Figure 7 shows the IRRAS reference measurement. IRRAS shows both volume and interfacial information about the PVAc due to the large depth of information. However, with the 20 nm film thicknesses chosen as reference, the information from the volume is significantly larger than from the interface. The peak structure for the reference measurement can be seen in Table 4 [40].

The assigned peaks show all components of the PVAc structure. The strong bands all belong to the acetoxy group (-OCOCH_3_) on the side chain. The measured weak bands all relate to the main carbon chain [40].

### 2.4. Characterization of the Interface between PVAc and Untreated or Plasma-Treated Aluminum

**Figure 8:**
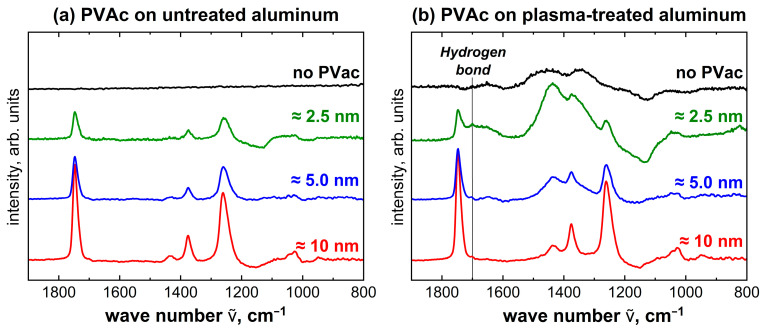
IRRAS measurement of 10 nm, 5 nm and 2.5 nm PVAc on (**a**) untreated aluminum and (**b**) plasma-treated aluminum. Shown in black are also the spectra of the uncoated aluminum surface.

To detect possible bonding mechanisms for untreated and plasma-treated aluminum with PVAc, measurements with decreasing film thickness were investigated again with IRRAS. Figure 8 shows the IRRAS spectra of PVAc film thicknesses of 10 nm, 5 nm and 2.5 nm each on untreated and plasma-treated aluminum. These thicknesses were chosen to detect changes in the interface. 

The IRRAS spectra for PVAc on untreated aluminum in Figure 8a show the same peaks as the reference measurement; only intensity and resolution decrease with reduction in film thickness. The spectra show no changes in the peak structure and no differences in the peak ratios relative to each other. Accordingly, the IRRAS measurements indicate no interaction between untreated aluminum and PVAc.

The same series of measurements was performed for the plasma-treated aluminum, which can be seen in Figure 8b. With decreasing film thickness, there is a significant change in the peak structure and in the peak intensity relative to each other. These changes to the PVAc reference can be caused either by the plasma treatment of the aluminum surface alone or by interactions between PVAc and aluminum. The most notable changes occur at 1432 cm^−1^ and 1364 cm^−1^. These peaks can be assigned to the OH and NO species, as already shown for the plasma-treated aluminum reference. Accordingly, this change in the peak structure can be explained by the plasma treatment of the aluminum surface only. A minor change occurs at 1650 cm^−1^, even for the 5 nm thick film. This can be explained by the plasma treatment of the aluminum surface as well since the species C=C can be assigned to this peak. At about 1700 cm^−1^, a low-intensity peak is formed, which is only visible at the lowest film thickness. This wave number cannot be assigned to either the plasma-treated aluminum surface or the PVAc. According to the literature [41], the peak at about 1700 cm^−1^ can be assigned to inter- or intramolecular hydrogen bonds. The observation that the signal occurs primarily in the thinnest films suggests that this intermolecular interaction is located at the interface between aluminum and PVAc. Accordingly, hydrogen bonds may form between the PVAc and the plasma-treated aluminum surface.

To confirm the results from the IRRAS measurements, a series of measurements with decreasing layer thickness was carried out with XPS. For both untreated and plasma-treated aluminum, PVAc concentrations of 0.25 wt.%, 0.1 wt.% and 0.025 wt.% were used, respectively. The intention of these low concentrations is to produce ultra-thin PVAc films of only 3 nm to 1 nm.

**Figure 9:**
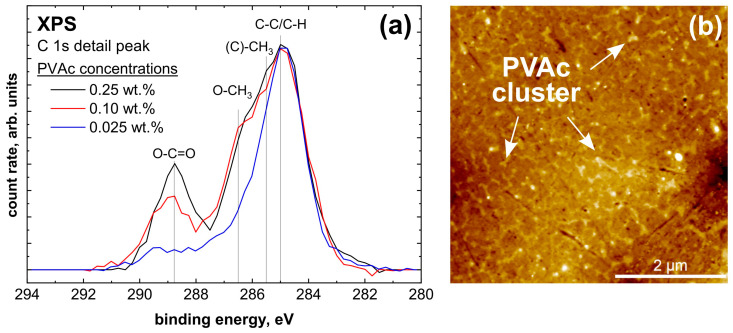
(**a**) Normalized XPS C 1s detail peaks with different PVAc concentrations in anisole on untreated aluminum and (**b**) AFM pictures of 0.1 wt.% PVAc on untreated aluminum.

The untreated aluminum sample, seen in Figure 9a, shows a clear change in the C 1s detail peak shape. For better comparability, the intensities of the spectra were normalized to the intensity of the C-C species. Both the O-C=O and O-CH_3_ species first decrease significantly at 0.1% and almost completely disappear at 0.025 wt.%. Accordingly, at the lowest concentration, the PVAc peak shape is no longer visible. Since the IRRAS measurements did not show any interaction between PVAc and untreated aluminum, and the undefined peak shape is more similar to atmospheric contamination, it can be assumed that the PVAc film is no longer closed and homogenous. AFM measurements were performed to confirm this assumption. In Figure 9b, a PVAc film with a concentration of 0.1 wt.% can be observed. In this image, the polishing ridges and unevenness of the aluminum sample can be seen. Contaminants are also visible, which can be attributed to storage and transport in the air atmosphere. These contaminants are a mix of PVAc and atmospheric impurities and do not originate from foreign metals or polishing residues. They consist primarily of C-C/C-H, C-O and C=O species, which can also be seen in Figure 4 for the untreated aluminum surface. Furthermore, it is clear that the film is not homogeneous and closed. The PVAc film appears to have contracted and formed clusters, as it is marked in Figure 9. This explains why the PVAc peak shape was no longer recognized in the XPS spectra. Accordingly, mainly impurities were measured, and no interactions between PVAc and untreated aluminum could be detected by XPS either.

**Figure 10:**
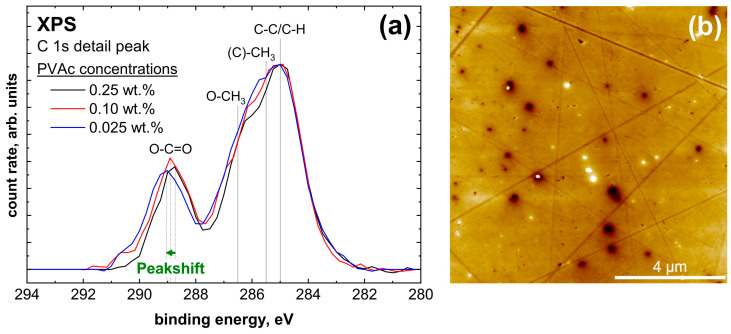
(**a**) Normalized XPS C 1s detail peaks with different PVAc concentrations in anisole on plasma-treated aluminum and (**b**) AFM pictures of 0.1 wt.% PVAc on plasma-treated aluminum.

For plasma-treated aluminum, a series of measurements with decreasing film thickness was also performed. This is shown in Figure 10a. The detail peak shows no significant change in the peak shape. Only a peak shift for the O-C=O species is visible. This species shifts from +4.10 eV for the reference to +4.25 eV for the thinnest film. Such a peak shift also indicates hydrogen bonding [42], as shown in the IRRAS measurement. Further, AFM measurements were performed, visible in Figure 10b, which show a homogeneous and closed film next to the polishing ridges, the unevenness and the impurities. 

## 3. Discussion

The results of Žigon et al. [13] showing that the plasma-treated aluminum and beech wood surfaces bonded with PVAc withstand significantly higher shear forces than untreated surfaces were explained on a molecular level in this work. Plasma treatment improves the surface properties of both the wood and the aluminum to interact better with the PVAc. In the case of beech wood, the proportion of polar species could be increased drastically, which presumably allows better bonding with the PVAc. Furthermore, the wettability should be increased through plasma treatment. 

The plasma treatment of the aluminum sample leads to a significant cleaning effect of the surface. Furthermore, there is also a clear activation of the surface with COOH and hydroxyl species, which leads to the formation of hydrogen bonds between the AL surface and the PVAc adhesive. For an untreated aluminum surface, both IRRAS and XPS showed no molecular interactions with the PVAc. This indicates that the PVAc may not strongly interact with the native aluminum oxide layer or the contaminations on the aluminum surface. An interaction through other weak intermolecular forces or mechanical interlocking cannot be ruled out, of course, and might explain the low shear strength that is nevertheless present. Plasma treatment of the aluminum sample allowed for hydrogen bonding to occur. Accordingly, it is likely that hydrogen bonding occurs between the carbonyl oxygen of the PVAc and the hydroxyl of the surface, which was significantly increased by the plasma treatment. Such interactions of hydrogen bonds between metal oxides and the carbonyl groups of similar polymers, such as PMMA or PECA, have also been shown in the literature [43,44,45]. Furthermore, hydrogen bonds with the COOH species would be conceivable. AFM measurements showed a closed and homogeneous film for the plasma treatment. As the PVAc adhesive is of a polar nature with its short ester side chain, the aluminum surface without plasma treatment provides an incompatible surface functionality with a negative impact on wetting processes. Accordingly, low concentrations of PVAc could not produce closed films without plasma treatment. In the case of plasma-treated aluminum, a large part of the surface serves as the contact area, which leads to many intermolecular interactions. This can also explain the increase in shear forces from 4.45 MPa for untreated aluminum to 7.54 MPa [13] for plasma-treated aluminum. Accordingly, PVAc could potentially replace more expensive adhesives like epoxy or polyurethane. These composites can be used in the construction industry, for example, with windows, but also for sports or music equipment.

## 4. Materials and Methods

### 4.1. Materials and Sample Preparation

Samples of anodized aluminum (AlMgSi05F22) and beech wood (*Fagus sylvatica* L.) were cut to dimensions of 10 × 10 mm^2^. The aluminum specimens were treated in a sanding machine (Jean Wirtz TG 250, Düsseldorf, Germany) with silicon carbide abrasive paper and, finally, diamond polished with a wool cloth. The result was a mirror-like surface finish. No leftovers from the polishing remained on the sample. Due to the preparation and storage in air, they contained a natural oxide layer on the surface. Prior to measurements and sample preparation, the aluminum samples were cleaned in an ultrasonic bath for 5 min each in isopropanol, acetone and distilled water. The trimmed wood samples were ground down to a thickness of 1 mm, and both sides were cleaned of coarse impurities using silicon carbide abrasive paper. Subsequently, they were stored in air until measurements were taken.

For the plasma treatment, a self-constructed DBD plasma setup was used under atmospheric air at a pressure of 1 atm. The plasma-treated area had a diameter of 12 mm. The treatment time was 10 s each time, and the distance between the plasma source and the aluminum sample was 3 mm, while for wood, it was 1 mm. The frequency used was 2.9 kHz (sinusoidal wave signal), and the voltage was set to 18 kV (peak-to-peak voltage). The duty cycle was 95%.

To investigate the molecular interactions between aluminum and PVAc, various polymer film thicknesses, ranging from 0.5 nm to 20 nm, were deposited using a commercial spin coater (SCI-30, Novocontrol, Montabaur, Germany). Each coating thickness was prepared twice, once with prior plasma treatment and once without any pre-treatment. The pure PVAc (Fisher Scientific, Schwerte, Germany) was dissolved in anisole (Sigma-Aldrich, Taufkirchen, Germany) at various concentrations for 24 h. Subsequently, the samples were cleaned with 40 µL of anisole in the spin coater and dried for one minute. Afterwards, the spin coating process was performed directly. For spin coating, 40 µL of the PVAc solution was used, and the sample was rotated at 83 rps for 60 s. Subsequently, the prepared samples were examined using IRRAS and XPS.

### 4.2. Characterization Methods

#### 4.2.1. Atomic Force Microscopy (AFM)

Using AFM (Cypher S, Oxford Instruments Asylum Research Inc, Santa Barbara, CA, USA), the morphology of the PVAc films on the aluminum substrate was investigated. These studies were performed in an air atmosphere and in tapping mode. Cantilevers with an aluminum coating (NSC15/Al BS, MikroMasch Europe, Wetzlar, Germany) were used. The cantilevers with dimensions of 125 × 30 × 4 µm^3^ have a force constant of 40 N/m and a resonance frequency of 325 kHz. The tip height is 15 µm with a full cone angle of 40°. The setpoint was adjusted to 0.6 V, and 512 lines were recorded with a scan rate of 1.5 Hz. The samples were examined after spin coating without further treatment. SPIP (version 6.1.1) was used as the evaluation program and to correct potential image errors.

#### 4.2.2. X-ray Photoelectron Spectroscopy (XPS)

The XPS examinations were carried out in a UHV chamber with a pressure of 10^−10^ mbar. The non-monochromatic AlK_α_ radiation used was generated by a commercial X-ray tube (RS40B1, Prevac, Rogów, Poland) with an energy of 1486.6 eV. The X-rays strike the sample at an angle of 80° to the sample normal. The emitted photoelectrons are detected at an angle of 10° to the sample normal with a hemispherical analyzer (EA10/100, Leybold, Köln, Germany). For the survey spectra, the pass energy was set to 80 eV, and the scan rate was 0.5 eV. In contrast, the detail spectra were recorded with a pass energy of 40 eV and a scan rate of 0.25 eV. These settings allow the individual peaks to be resolved more precisely. These spectra were used to determine the stoichiometry of the individual elements present, which is done by evaluating the individual peak areas. The evaluation included the sensitive factors of Scofield as well as the transmission function of the analyzer and the angle between the X-ray tube and the analyzer. The results of the measurements were evaluated using CasaXPS software. A Shirley background correction was performed, and a Gauss–Lorentz mixture was used as the peak shape for deconvolution of the peak signal. The peak fit was performed with the Levenberg–Marquardt algorithm. The observed charging effects were corrected by setting the aliphatic carbon peak to 285 eV. In order to prevent or reduce radiation damage, the C 1s carbon peak was recorded first, followed by the other detail peaks.

To determine the layer thickness of the PVAc film on the aluminum surface, the peak areas of the Al 2p peak of the aluminum reference sample *I_0_* are compared with the peak area of the coated samples *I_x_*. The following formula is used for this purpose [46]:

$$d=\lambda \left(E\right)\cdot \cos\left(\alpha \right)\cdot \ln\left(\frac{I_{0}}{I_{x}}\right)$$

The angle α refers to the detection direction and corresponds to 10°. The Al 2p electrons have a kinetic energy of 1414 eV; accordingly, a value of 4.40 nm is given for λ [47]. Due to a lack of literature on the free path length of the electrons in the PVAc, the value of the chemically similar PMMA is used.

#### 4.2.3. Infrared Reflection Adsorption Spectroscopy (IRRAS)

For the investigation of the aluminum samples by means of IRRAS, the measurements were performed with a commercial A513Q variable angle reflection accessory for a vertex 70V FTIR spectrometer (Bruker, Bremen, Germany). The examinations were carried out in a spectral range of 400 cm^−1^ to 4500 cm^−1^ with a resolution of 2 cm^−1^. P-polarized IR light was used, which hits the sample at an angle of 80° to the surface normal. For each sample, 100 measurements were recorded and averaged. Each recorded measurement was based on a reference measurement of a cleaned aluminum sample, which was recorded first.

## Figures and Tables

**Table 1:** Area fractions of binding species in the XPS C 1s detail peak for untreated and plasma-treated beech wood.

Species	Untreated Beech Wood	Plasma-Treated Beech Wood
C-C/C-H	74.7%	33.2%
C-O	19.6%	43.3%
O-C-O	3.5%	9.5%
O-C=O	2.2%	14.0%

**Table 2:** Area fractions of binding species in the XPS C 1s detail peaks for untreated and plasma-treated aluminum.

Species	Untreated Aluminum	Plasma-Treated Aluminum
C-C/C-H	64.4%	62.8%
C-O	16.2%	20.3%
C=O	19.4%	-
COOH	-	16.9%

**Table 3:** Position and area fractions of binding species in the XPS C 1s detail peak for the PVAc reference.

Species	Position	FWHM	Proportion
Aliphatic C-C/C-H	285.00 eV	1.65 eV	25.1%
(C)-CH_3_	285.46 eV	1.75 eV	24.9%
O-CH_3_	286.66 eV	1.67 eV	25.4%
O-C=O	289.10 eV	1.45 eV	24.6%

**Table 4:** IRRAS peak structure for the PVAc reference.

Wave Number	Species
1748 cm^−1^	C=O (symmetric stretching mode)
1436 cm^−1^	C-H_2_ (deformation mode)
1376 cm^−1^	C-H_3_ (deformation mode)
1261 cm^−1^	C-O-C (antisymmetric stretching mode)
1095 cm^−1^	C-H_2_ (wagging mode)
1025 cm^−1^	C-O-C (symmetric stretching mode)
947 cm^−1^	C-H

## Data Availability

All datasets, including raw and analyzed data, used for this publication are available at https://doi.org/10.5281/zenodo.8385402. URL (accessed on 6 November 2023).

## Supplementary Materials

The following supporting information can be downloaded at: https://www.mdpi.com/article/10.3390/molecules28227574/s1. Figure S1: Survey spectrum of untreated and plasma-treated beech wood; Figure S2: Survey spectrum of untreated and plasma-treated aluminum; Figure S3: O 1s detail spectra of plasma-treated aluminum; Figure S4: N 1s detail spectra of plasma-treated aluminum.
