# Supplementary material for: Characterization of Molecular Interactions in the Bondline of Composites from Plasma-Treated Aluminum and Wood
Source: Molecules. 2023 Nov 14;28(22):7574. doi: 10.3390/molecules28227574 (PMC10673544; doi:10.3390/molecules28227574)
Supplement: Supplementary file 1 [file molecules-28-07574-s001.zip › molecules-2663485-supplementary.pdf]

Supporting Information

Characterization of Molecular Interactions in the Bondline of Composites from Plasma-Treated Aluminum and Wood

Sascha Jan Zimmermann [1], Philipp Moritz [1], Oliver Höfft [2,*], Lienhard Wegewitz [1], Wolfgang Maus-Friedrichs [1] and Sebastian Dahle [3]

[1] Clausthal Center for Materials Technology, Clausthal University of Technology, Agricolastrasse 2, 38678 Clausthal-Zellerfeld, Germany
[2] Institute for Electrochemistry, Clausthal University of Technology, Arnold-Sommerfeld-Strasse 6, 38678 Clausthal-Zellerfeld, Germany
[3] Department of Wood Science and Technology, Biotechnical Faculty, University of Ljubljana, Jamnikarjeva Ulica 101, 1000 Ljubljana, Slovenia
* Correspondence: oliver.hoefft@tu-clausthal.de

1. Supporting Information

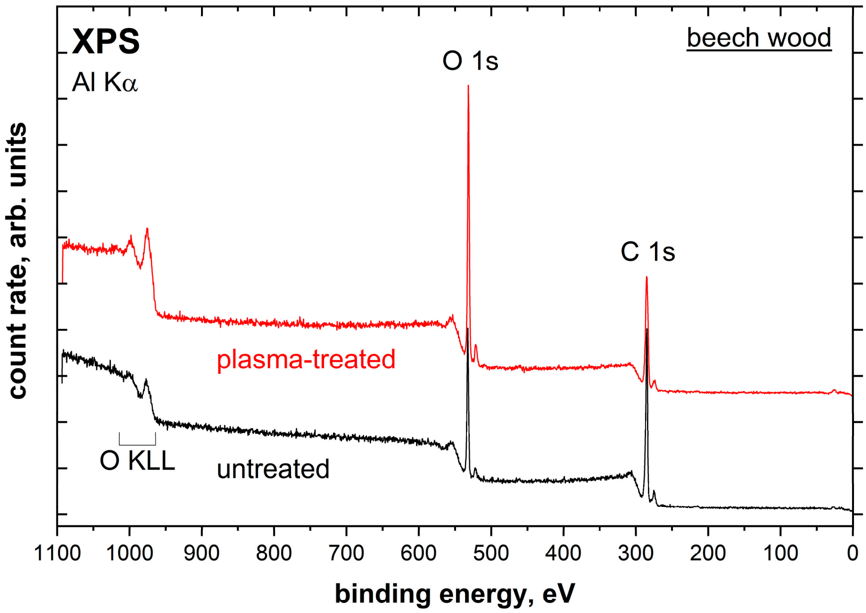

Figure S1. Survey spectrum of untreated and plasma-treated beech wood

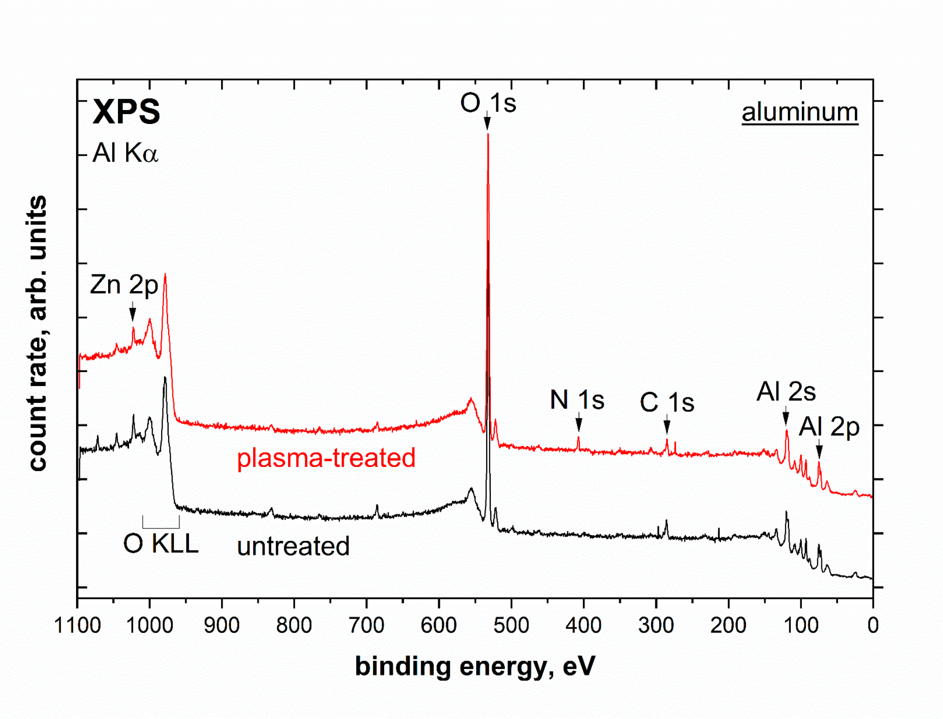

Figure S2. Survey spectrum of untreated and plasma-treated aluminum

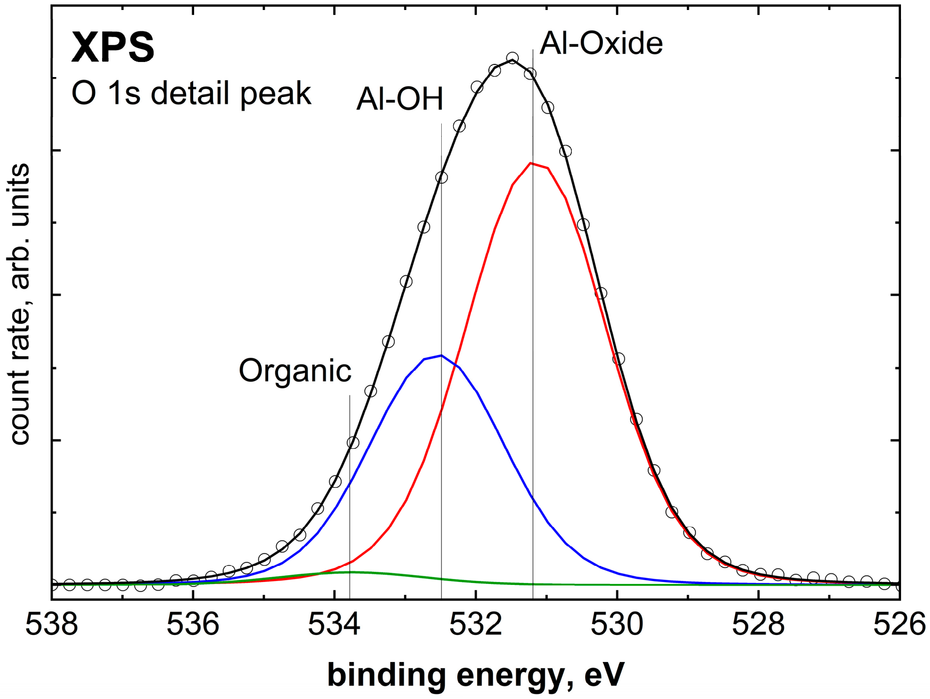

Figure S3. O 1s detail spectra of plasma-treated aluminum

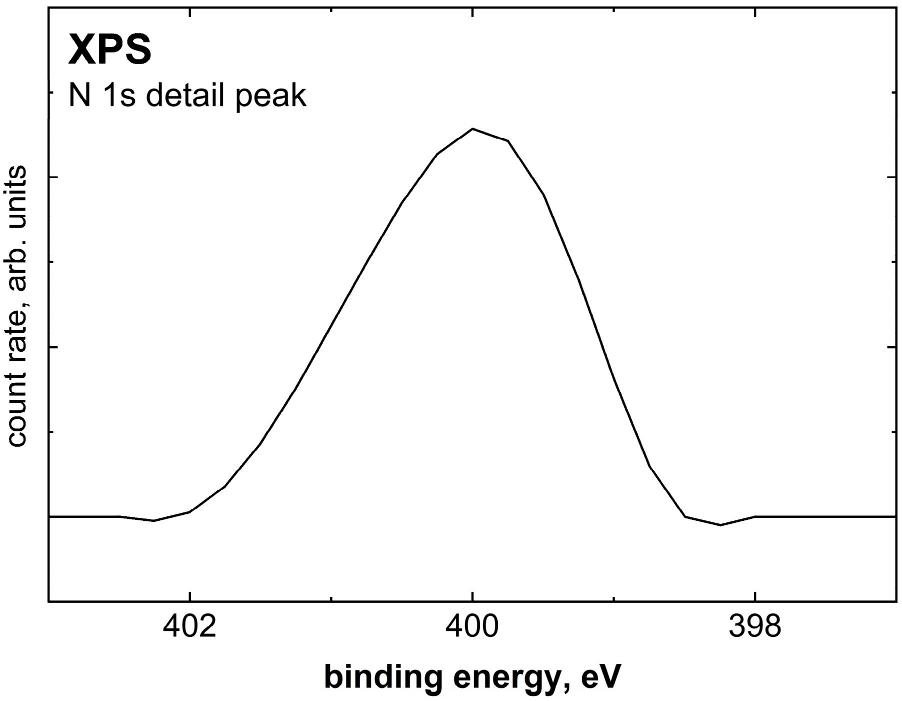

Figure S4. N 1s detail spectra of plasma-treated aluminum
